# Fostering Intergenerational Relationships at U.S. Men’s Sheds

**DOI:** 10.1093/geroni/igaf122.2761

**Published:** 2025-12-31

**Authors:** Melinda Heinz, Katie Cullen

**Affiliations:** University of Northern Iowa, Cedar Falls, Iowa, United States; University of Northern Iowa, Cedar Falls, Iowa, United States

## Abstract

Men’s Sheds began in Australia during the 1990’s and they now have a presence in eight other countries, including the U.S. The purpose of this study was to investigate intergenerational relationships at one of the only known intergenerational sheds in the U.S. Ten members participated in interviews about their experiences with Men’s Sheds. Interviews were audio recorded and transcribed verbatim. Braun and Clark’s (2020) six-step thematic approach were utilized to analyze the transcripts. Steps include data familiarization, coding, early theme generation, refining themes, paring down themes, and results write up. Results revealed, Theme 1: Intergenerational Transmission and Generativity, represented by older members commenting on a sense of purpose when sharing advice or perspectives with younger members. “There’s a purpose for us being here and that is to help each other…there’s several men that we are able to just share things with.” Another member said, I would rather be here doing whatever needs doing encouraging other people than doing anything else.” Younger members appreciated the guidance with learning how to use tools or saws in the woodshop, commenting, “So I’ve learned pretty well how to use everything over there.” Theme 2: Community Outreach represented the community partnerships members created with youth organizations ranging from “bringing the Cub Scouts out” to helping home schooled children “build birdhouses.” These findings indicate that there is potential to build meaningful intergenerational relationships at Men’s Sheds that are mutually beneficial, providing a purposeful outlet for older adults and teaching younger adults practical skills such as woodworking.

